# Factors affecting performance and manufacturability of naproxen Liqui-Pellet

**DOI:** 10.1007/s40199-020-00362-9

**Published:** 2020-08-05

**Authors:** Matthew Lam, Ali Nokhodchi

**Affiliations:** Pharmaceutics Research Laboratory, Arundel Building, School of Life Sciences, University of Sussex, Brighton, UK

**Keywords:** Liqui-Pellet, Liqui-Mass system, Naproxen, Solid-state analysis, Dissolution enhancement, Liquisolid technology

## Abstract

**Aim:**

Liqui-Pellet is potentially an emerging next-generation oral pill, which has shown promising results with unique advantages as well as displaying potential for commercial feasibility. Since Liqui-Pellet technology is still in its infancy, it is important to explore the parameters that can affect its performance, particularly the drug release rate. Therefore, the aim of this study is to investigate thoroughly the effect of Avicel PH101 (carrier) and Aerosil 300 (coating material) ratio (R-value) in Liqui-Pellet.

**Methods:**

Key parameter for Liqui-Pellet formulation in this study was the ratio of carrier and coating material. Tests were carried out to assess the physicochemical properties of different formulations. This involved looking into particle size, robustness, flowability, solid-state and drug release profile. The morphology of Liqui-Pellet was investigated by SEM.

**Results:**

It is found that R-value does not have a major effect on the success of Liqui-Pellet production. However, R-value does seem to have an effect on Liqui-Pellet size at a certain water content level and a slight effect on the drug release rate. A decrease in Avicel PH101 concentration and an increase in Aerosil 300 concentration in Liqui-Pellet formulations can reduce Liqui-Pellet size and slightly increase drug release rate by 9% after 2 h. The data shows Liqui-Pellet is resistant to friability, able to achieve exceptional flow property and have smooth surfaces, which is critical for applying coatings technology. Such properties are ideal in terms of commercial manufacturing. The XRPD and DSC both show the reduction in formulation crystallinity, which is expected in Liqui-Pellet formulation as a result of solubility of the drug in the co-solvent used in the preparation of Liqui-Pellets.

**Conclusion:**

Overall it seems that R-value can affect Liqui-Pellet drug release rate and size but not on the production success rate.

**Graphical abstract:**

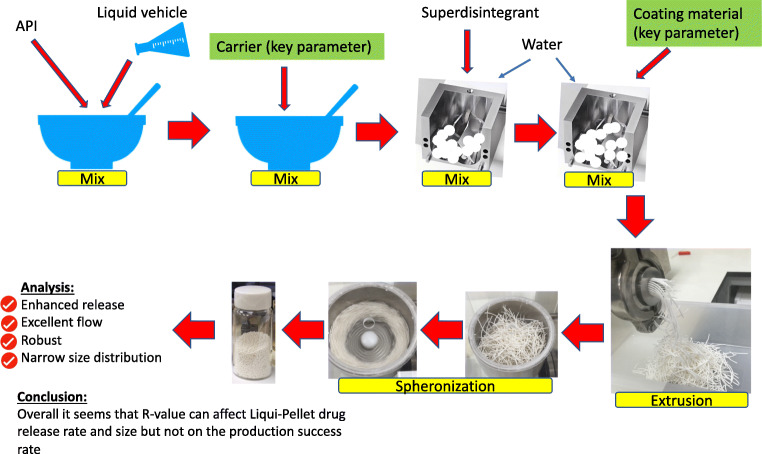

## Introduction

Liqui-Pellet is an emerging novel oral dosage form, which focuses on tackling the challenges of developing future oral drug delivery system. The challenges include improving drug efficacy and safety whilst ensuring cost-effectiveness. Liqui-Pellet aims at improving drug efficacy through improving bioavailability, particularly poorly water-soluble active pharmaceutical ingredient (API), via enhancing drug dissolution rate [1–3]. Poor bioavailability due to the poor drug release rate of such API is a well-known challenge in the pharmaceutical industry. Enhancement of the bioavailability of these API can potentially reduce adverse drug effects by potentially lowering dosage strength. It can also potentially bring more viable API to the market through achieving acceptable bioavailability that would otherwise have not been acceptable. The poor bioavailability of water-insoluble drugs is mainly due to poor drug dissolution rate [4]. Liqui-Pellet is capable of enhancing the drug release rate whilst achieving excellent flow properties and high load factor unlike the classical liquisolid compact [1–3]. This makes the next generation Liqui-Pellet formulation highly commercially feasible, unlike in the classical liquisolid technology. Details of enhancing drug release mechanisms are similar to liquisolid formulation, which can be found in various literature [5–9]. In brief, it is postulated that the enhanced drug release rate is due to an increase in surface area available for dissolution, increased solubility of the drug and improved wettability of drug particles [5, 6].

Liqui-Pellet formulation is mainly targeted at BCS (biopharmaceutical classification system) class II drugs in order to enhance their bioavailability. This is because it is usually the drug dissolution rate that is the rate-limiting step for bioavailability in these drugs [1–3]. Approximately 60% of drugs that are commercially available and 40% [10, 11] or 90% [12] (depending on different sources) of drugs in developing pipelines are identified as poorly water-soluble, which is based on BSC.

Liqui-Pellet stems from liquisolid concept and pelletization technologies, however, it is different from liquisolid technology in that Liqui-Mass system is used instead of liquisolid system. In liquisolid system, an admixture of API and excipients are free-flowing powder, whereas Liqui-Mass system is usually a wet mass [1, 2]. The wet mass system can give a degree of versatility in the technology because excipients are not restricted due to flowability, which is seen in the classical liquisolid technology. In a pellet form, there is even more versatility such as the application of coating technology and the inherent flexibility in controlled delivery system in multi-dose unit.

It is claimed by the authors that Liqui-Pellet is considered to be ideal for commercial production because it is simple (no advance machine or technique is required), cost-effective (materials used are common and easily obtainable) and uses green technology [2, 4, 5]. The production of Liqui-Pellet is considered to be easily up-scaled because the types of machinery and excipients required are typically available in pharmaceutical manufacturing facilities. Liqui-Pellet can be made in such a way that explosive or sustained drug release can be achieved. In unpublished data, the pH at the microenvironment can even be modulated to improve the drug release rate [13, 14]. In addition, Liqui-Pellet produced from previous studies [1–3, 13, 14] have shown promising results in terms of commercial production and quality control such as, resistance to friability, narrow size distribution and smooth surface structure, which is ideal for coating. Coating Liqui-Pellet has major implications such as extended, delayed and gastro-retentive release, as well as taste masking for pediatric use.

In the authors’ previous studies, it is shown the crucial effect of water and Tween 80 (liquid vehicle) on modulating the drug dissolution rate in Liqui-Pellet [3]. In brief, an increase in Tween 80 concentration and a decrease in water content improves drug release rate [3]. With this knowledge, it is possible to optimize and tailor the drug release profile; hence, this fundamental knowledge is imperative in formulating Liqui-Pellet.

Aerosil 300 is a colloidal silicon dioxide, which is one of the main coating material used in Liqui-Pellet production. It is also the coating material used in this study. Other than the coating function, Aerosil has applications such as adsorbent, tablet disintegrant, thermal stabilizer and viscosity increasing agent [15]. The hygroscopic Aerosil 300 is capable of adsorbing large quantities of water without being liquefied [15]; this allows it to scavenge excess water during the granulation process in Liqui-Pellet production. Since colloidal silicon dioxide can also be used as a tablet disintegrant [16], it may have an effect on the disintegration of Liqui-Pellet, effecting the drug release rate, which this study will be investigating.

The main carrier used in this investigation is Avicel PH101, which is microcrystalline cellulose (MCC). MCC is the gold standard carrier in extrusion-spheronization technology due to its unique rheological properties, cohesiveness and plasticity in order to yield robust spherical pellets [17]. However, one of the major limitations of using MCC in pelletisation is the strong bonding, rendering the pellet none disintegrating [18].

On the basis of the above description, as Liqui-Pellet is a new emerging technology, there are still so many parameters affecting its performance which should be explored. Therefore, the aim of the investigation is to look at the effect of carrier and coating material ratio.

## Materials and methods

### Materials

Naproxen was purchased from Tokyo Chemical Industry Co (TCI, Japan). Colloidal silicon dioxide (Aerosil 300), (Evonik Industries AG, Hanau, Germany); microcrystalline cellulose (Avicel PH-101), (FMC corp., UK); sodium starch glycolate Type A (Primojel), (DFE Pharma, Goch, Germany) and polysorbate 80 (Tween 80), (Acros, Netherlands) were used. All other reagents and solvent were of analytical grades.

### Production of naproxen Liqui-Pellet

The preparation of Liqui-Pellet is carried out in a similar manner as in the authors’ previous work on Liqui-Pellet [1–3]. It involved solubilizing specified amount of naproxen in a specified amount of Tween 80 (non-volatile co-solvent) using pestle and mortar. Once naproxen was thoroughly mixed and solubilized in the liquid vehicle, a pre-determined quantity of Avicel PH101 (carrier) was added and further mixed. The admixture of wet mass was then transferred into a blender (Caleva Multitab, Caleva Process Solutions Ltd., UK), where disintegrant (Primojel) with a concentration of around 5% w/w was added to the blend to improve disintegration of the formulation. The wet mass admixture was then mixed under a constant rate of 125 rpm for 7 min with a specified quantity of deionized water incorporated gradually. At this point, Aerosil 300 (coating material) was added into the wet mass and further undergo another 7 min of mixing. The wet mass was then processed to make extrudates where it is then placed on a spinning frictional plate at an almost constant rotation of about 4000 rpm. It should be noted that for each formulation, spheronization time varied depending on the extrudate physical property. The key parameters that were varied were water content, Tween 80 concentration and R-value. Some formulation extrudates were treated in the freezer, liquid nitrogen or were sphereonized in small quantity at a time in an attempt to increase the success rate of producing Liqui-Pellet. Details of each formulation can be seen in Table 1. Note that Tween 80 was chosen as the liquid vehicle in this investigation because based on the authors’ previous studies, it is currently the most suitable liquid vehicle [3].

**Table 1:** Key formulation characteristics of the investigated Liqui-Pellet capsule

Formulation	Water content during extrusion-spheronization (ml) per 20 g of admixture of API and excipients	Amount of Tween 80 (% w/w)	Additional treatment	Liquid load factor ^a^	Mass of Avicel PH101 (mg)	Mass of Aerosil 300 (mg)	R-Value ^b^	Successfully spheronized into pellets? (Yes/No)	Total weight of 25 mg naproxen Liqui-Pellet (mg)
Physical mixture pellet	12				58.15	2.90	20:1	Yes	90.58
LP-1	8.57	36		1.38	49.58	5.22	19:2	No	
LP-2	4.76	40		1.63	45.07	4.74	19:2	No	
LP-3	4.76	40	Frozen in freezer	1.63	45.07	4.74	19:2	No	
LP-4	1.90	40		1.63	45.07	4.74	19:2	No	
LP-5	1.90	40	Frozen in freezer	1.63	45.07	4.74	19:2	No	
LP-6	1.90	40	Spheronized little by little	1.63	45.07	4.74	19:2	Yes	131.25
LP-7	0.95	36		1.38	49.58	5.22	19:2	No	
LP-8	0.95	36	Treated with liquid nitrogen	1.38	49.58	5.22	19:2	No	
LP-9	4.76	36		1.38	52.77	2.64	20:1	Yes	132.70
LP-10	4.76	36		1.38	49.58	5.22	19:2	Yes	131.25
LP-11	4.76	36		1.38	44.36	7.83	06:1	Yes	128.64
LP-12	1.90	36		1.38	52.77	2.64	20:1	Yes	132.70
LP13	1.90	36		1.38	49.58	5.22	19:2	Yes	131.25
LP-14	1.90	36		1.38	44.36	7.83	06:1	Yes	128.64

Note that all formulation contained 25 mg of naproxen, and Primojel ~5% *w*/w

^a^Liquid load factor is the weight ratio of the liquid medicine and carrier

^b^R-Value is the ratio of the carrier and coating material

### Evaluation of formulated Liqui-Pellet

#### Analysis of pellet size using sieve method

Particle size analysis of all successful formulations (except for LP-6 as its surface was too cohesive/sticky for this test) was done using sieves and mechanical shaker method. Specified formulation weighing 5 g was placed in a sieve (Test sieve, Retsch, Germany). The sieve sizes that were used were 2000, 1000, 850, 500 and 250 μm. The sieve then placed on a mechanical shaker (AS 200, Retsch, Germany) was set under two different vibration amplitudes (first 1 min vibrated under 60 amplitudes followed by 9 min under 40 amplitudes). The collected fractions were weighed and size distribution was constructed for each formulation.

#### Analysis of formulation robustness using friability test

The robustness of the produced formulation was assessed using the friability test. This test was carried out to all successfully produced formulation, except for LP-6 as its surface was too cohesive/sticky for this test. Chosen formulation of Liqui-Pellet weighing 3 g was placed in a friabiliator (D-63150, Erweka, Germany) with 3 g of glass beads. The opening of the friabilator chamber was closed in order to stop pellets falling out of the friabilitor chamber. The friabilator was then set under constant rotation of 25 rpm for 4 min. Basic percentage calculation was applied to determine the percentage weight loss of the sample.

#### Tests on the flow property of successful formulations

All successfully produced formulation underwent flowability test (except for LP-6 as its surface was too cohesive/sticky for this test). Three flowability tests were carried out, which includes the angle of repose (Copley Scientific, UK and Digimatic height gage, Mitutoyo, Japan), flow rate in g/s (Flowability tester, Copley Scientific, UK), and Carr’s compressibility index using tapped density tester (D-63150, Erweka, Germany). The angle of repose was determined by placing a specified amount of Liqui-Pellet formulation in a funnel with 10 mm diameter opening and let to flow onto a test platform. Then the height and diameter of the heap of the sample were measured in order to calculate the angle of repose. Flow rates were determined by recording the sample weight (g) and time (sec) of the sample flowing through a 10 mm diameter opening. Bulk density was measured using a measuring cylinder and tapped density was determined using the tapped density tester (tapped for exactly a hundred times). The bulk and tapped densities were used to calculate Carr’s compressibility index (CI% = [Tapped density - bulk density/tapped density] × 100). All measurements were done in triplicates.

#### Scanning electron microscope (SEM) analysis

The surface structure of all successfully produced formulations was analyzed using SEM (Jeol JMS 820, Freising, Germany). Double-sided carbon tape containing Liqui-Pellet formulations was sputter-coated with gold using a sputter coater (Edwards S-150 sputter coater, Edwards High Vacuum Co. International, USA) with gold target and Argon gas under 5 kV for 5 min. The sputter-coated sample was then placed in the SEM machine where the Liqui-Pellet surface structure was observed and recorded at 80 times and 800 times magnifications operating at 3 kV.

### In-vitro drug release test

Dissolution tests were performed on all successfully produced formulations via USP apparatus 2 (708-DS Dissolution Apparatus & Cary 60 UV-Vis, Agilent Technologies, USA), using the same parameters and amount of API as in previous studies (1,2). Capsules of a specified formulation containing 25 mg of naproxen underwent dissolution. The set condition in the dissolution test includes 900 ml dissolution medium, temperature of 37.3 ± 0.5 °C and paddle agitation of 50 rpm. The pH of the dissolution medium used was either pH 1.2 or pH 7.4 (the dissolution medium was made according to USP pharmacopoeia), which simulate the gastrointestinal fluid with the absence of enzymes. Spectrophotometric analysis was set to absorbance wavelength at 271 nm. The absorbance readings were taken at 5, 10, 15, 20, 25, 30, 35, 40, 45, 50, 55, 60, 70, 80, 90, 100, 110 and 120 min.

Difference factor (f_1_) and similarity factor (f_2_) were used to compare dissolution profiles for various formulations [19]. FDA has also recommended using f1 and f2 for comparison of two dissolution profiles [20–22]. In brief, the two dissolution profiles are considered similar if f_1_ value is less than 15 or f_2_ value above 50 [23].

### Differential scanning calorimetry (DSC) and X-ray powder diffraction (XRPD) studies

Solid-state studies were performed using DSC (DCS 4000, Perkin Elmer, USA) and XRPD (D5000, Siemen, Germany). Both DSC and XRPD were performed on the drug and each solid excipient used in the preparation of Liqui-Pellet, physical mixture pellet and all successfully produced Liqui-Pellet formulations except for LP-6 as it is too plastic and soft to be crushed into powder form for XRPD. DSC was carried out by placing 3–6 mg of the sample in an aluminium pan where the edges were then crimped to seal the pan. The sealed pan was then placed in the DSC chamber under nitrogen atmosphere where scanning rate was 10 °C/min from 25 °C to 200 °C. The thermal behaviour correlates to the solid-state of the drug (amorphous or crystalline).

XRPD was carried out at scanning angle ranged from 5^o^ to 40^o^ with a scan rate of 0.02^o^/s at a voltage of 40 kV and current of 30 mA. The crystalline peak of naproxen at 18.9^o^ was used to compare crystallinity among the different formulations. Two methods were used to calculate the percentage relative crystallinity, which was an integrated peak area and peak height methods. For the integrated peak area method, a software called PANalytical X’Pert Highscore Plus was used to determine the area under the specified peak.

## Results and discussion

### Production of Liqui-Pellet formulation

Successful and unsuccessful formulations can be seen in Table 1. Only formulation LP-6 and LP-9 to LP-14 were successfully made into Liqui-Pellet. The rest of the Liqui-Pellet formulations agglomerated during the spheronization process due to extrudate surface exhibiting a critical degree of cohesive property. As seen in Table 1, there was a range limit in the amount of water and Tween 80 concentration, which determined whether Liqiui-Pellets could be produced successfully. When the water content was high, which could be seen in LP-1 (8.57 ml of water per 20 of Liqui-Mass admixture), agglomeration occurred during the spheronization process. Agglomeration also occurred when the water content was too low, which can be seen in LP-7 and LP-8 (0.95 ml of water per 20 g of Liqui-Mass admixture). When the concentration of Tween 80 as high as 40% w/w, all formulation agglomerated except for LP-6. This indicates the limit of water and liquid vehicle content in the formulation. According to previous studies by the authors, the limitation of water and Tween 80 was due to the plastic properties that these two parameters contributed to [3]. Extrudate should be plastic enough to form spherical pellets when spheronized but not too plastic or soft that would result in agglomeration. In addition, the Tween 80 enhances the cohesiveness of the extrudate surface, which increases the likelihood of agglomeration.

The ratio of carrier to coating material does not seem to have an effect on the success of the Liqui-Pellet production. This is a surprise as it is initially thought that increasing Aerosil 300 would reduce the risk of agglomeration during spheronization process due to an increase of dry property. Formulations LP-9 to LP-14 all have different R-value but were able to be spheronized into Liqui-Pellet. It seems like only the water and the liquid vehicle parameters have the greatest influence on the success of Liqui-Pellet production, which is further supported in the authors’ previous studies [3].

It is worth mentioning that some of the failed formulation’s extrudates misleadingly seemed ideal for spheronization, but in fact, agglomerated when spheronized. For example, LP-7 extrudate was brittle and short in length, which would seem ideal for spheronization; however, during the actual spheronization process the sample agglomerated due to a sticky surface. Perhaps with a small amount of water and a high amount of liquid vehicle, the extrudate is prone to the leakage of the liquid vehicle from the core of extrudates to the surface, causing the agglomeration.

An attempt was made to succeed in the failed formulation by freezing the extrudate via freezer (LP-3 and LP-5) or liquid nitrogen (LP-8) before spheronization process in an attempt to increase the brittleness of the extrudate. However, the attempts were unsuccessful in producing pellets. All formulation containing a high amount of Tween 80 (40% w/w) agglomerated during spheronization process except for LP-6. The agglomeration was avoided and Liqui-Pellet was produced successfully by spheronizing a small amount of extrudate bit by bit whilst constantly adjusting the spheronization speed. This shows that the success of Liqui-Pellet production outside the water and liquid vehicle typical limit can be achieved by adjusting the production parameters such as extrudate load and spheronization speed.

### Particle size of formulated Liqui-Pellet via sieve method

**Fig. 1:**
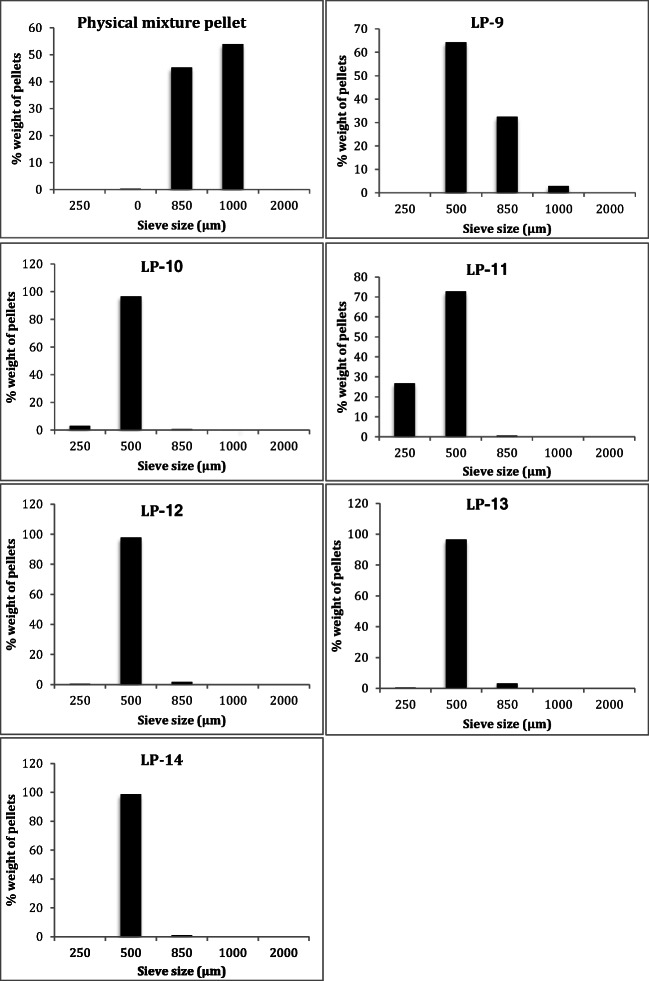
Particle size distribution of physical mixture pellet and formulation LP-9 to LP-14

Formulations LP-9, LP-10 and LP-11 contained 4.76 ml of water per 20 g of Liqui-Mass admixture and 36% w/w Tween 80 (Table 1); however, the R-values was different. In these formulations, there seems to be a trend where decreasing R-value resulted in a higher proportion of smaller size Liqui-Pellet (Fig. 1). In formulation LP-9 (R-value = 20:1), ~64% of Liqui-Pellets was within 500 μm and 0% was within 250 μm. Formulation LP-10 (R-value = 19:2) have a higher proportion of smaller Liqui-Pellet than LP-9, where ~97% of Liqui-Pellets was within 500 μm and 3% was within 250 μm. As the R-value further decreased to 6:1 as seen in LP-11, there was a further increase in the proportion of smaller size Liqui-Pellet (73% within 500 μm and 27% within 250 μm). It seems that the ratio of carrier to coating material may have some influence on Liqui-Pellet size. Such a trend of decreasing pellet size on decreasing R-value is not observed for formulation containing 1.9 ml of water per 20 g Liqui-Mass admixture and 36% *w*/w Tween 80 (LP-12, LP-13 and LP-14). This could indicate that when a small amount of water content is used, R-value loses its influence on Liqui-Pellet size. Also, there is a possibility that spheronization speed and duration time could have an effect on the Liqui-Pellet size, however, such parameter is outside the scope of the current investigation but is noteworthy for future investigation.

### Studies of robustness via friability test

In accordance with USP, friability test for tablets are considered acceptable if less than 1% weight loss is observed. Since pellets do not have a standard for friability test, this USP standard for tablets is adapted in the friability study. All successful formulations except LP-6 underwent friability test and have a weight loss of less than 1% (Table 2), indicating that the Liqui-Pellet is robust enough in regard to commercial production. In general, Liqui-Pellets are known to be robust, which is supported in the authors’ previous friability studies [1–3]. Note that LP-6 surface is too cohesive to undergo friability test. The pellet would stick into the machine rendering the test unsuitable.

**Table 2:** Weight loss of 3 g of each formulation under rotational speed of 25 rpm for 4 min

Formulation	% Weight loss
Physical mixture pellet	0.14
LP-9	0.09
LP-10	0.45
LP-11	0.42
LP-12	0.29
LP-13	0.46
LP-14	0.63

The MCC (carrier) in the formulation gives Liqui-Pellet strong bonding within its structure; in addition, the Tween 80 gives the Liqui-Pellet its plastic and soft properties. Both of these contribute to its resistance to friability.

### Flow properties

Physical mixture pellet and all successful Liqui-Pellet formulations, except for LP-6, underwent flowability test. LP-6 Liqui-Pellet surface was too sticky for flowability testing. All other successful formulations were subjected to flowability testing and showed excellent, excellent-good or good-fair flow properties (Table 3). This result further supports Liqui-Pellet technology ability to overcome the drawback of poor flow property as seen in the classical liquisolid formulation, rendering it ideal for commercial manufacturing as the flow property would allow reliable filling of capsules. It is worth stating that these formulations have a high liquid load factor. The unique high liquid load factor without issue regarding flowability makes Liqui-Pellet an interesting product. It also makes Liqui-Pellet simpler than liquisolid formulation by not having to rely on the liquisolid mathematical model that was introduced by Spireas [1–3].

**Table 3:** Flow rate (g/s), Angle of repose and Carr’s compressible index (CI%) of all formulations (*n* = 3)

Formulation^a^	Flow Rate (g/s) ± SD^b^	Angle of repose ± SD^b^	CI% ± SD^b^	Inference according to Angle of repose	Inference according to CI%
Physical mixture pellet	10.72 ± 0.33	19.96 ± 1.43	11.11 ± 0.62	Excellent flowability	Good flowability
LP-9	7.07 ± 0.11	27.65 ± 1.00	6.31 ± 0.70	Excellent flowability	Excellent flowability
LP-10	5.28 ± 0.06	32.74 ± 0.40	6.38 ± 1.20	Good flowability	Excellent flowability
LP-11	3.96 ± 0.18	36.51 ± 0.95	8.44 ± 1.21	Good-fair flowability	Excellent flowability
LP-12	6.4 ± 0.19	29.52 ± 0.85	3.96 ± 0.00	Excellent flowability	Excellent flowability
LP-13	6.10 ± 0.09	29.29 ± 0.50	4.48 ± 1.27	Excellent flowability	Excellent flowability
LP-14	6.09 ± 0.18	30.35 ± 0.58	6.01 ± 1.14	Excellent-good flowability	Excellent flowability

^a^For the composition of each formulation refer to Table 1

^b^SD, standard deviation from the mean

### Studies of surface structure via SEM

**Fig. 2:**
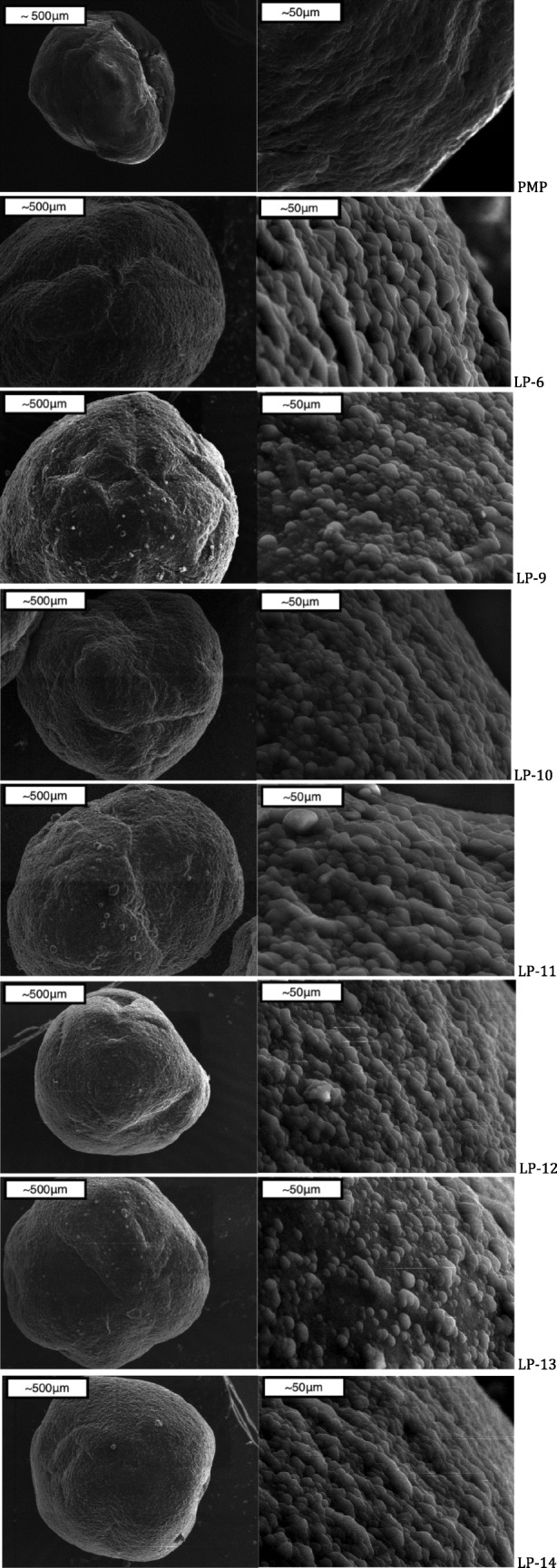
SEM images of physical mixture pellet and all successful formulation; I. × 80 magnification and II. × 800 magnification

In comparison with physical mixture pellet and successful formulations, it can be clearly seen that Tween 80 has a significant impact on Liqui-Pellet surface morphology (Fig. 2). All of the successful formulations have a smooth pebble-like surface, unlike the physical mixture. It seems like a formulation with the highest concentration of Tween 80 such as LP-6 (40% w/w) has slightly steeper pebble-like surface structure than formulations containing 36% w/w Tween 80 (LP-9 to LP-14). All of the successful formulations containing 36% w/w Tween 80 have similar surface morphology among each other. However, there is a slight variation in the surface structure, which could be due to the results of inconsistence spheronization speed and duration. These two parameters were subjected to constant adjustment to achieve successful Liqui-Pellet production, which is the priority of the investigation.

As seen in Fig. 2, all formulations have relatively smooth surface morphology. This is critical for applying coating [24], which is an important factor when considering extended, delayed, controlled and gastro-retentive release formulation. Also, since the Liqui-Pellets have a smooth spherical shape, it would be easier to apply taste masking polymer [25–28], which have implication for pediatric formulation.

### Drug dissolution studies

**Fig. 3:**
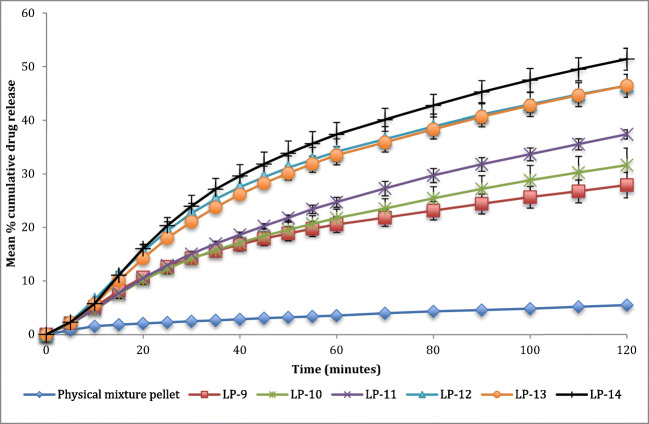
Dissolution profile of all successful Liqui-Pellet formulation containing 25 mg of naproxen in capsule (pH 1.2)

It can be seen in Fig. 3 that formulation containing 1.9 ml of water per 20 g of Liqui-Mass admixture (LP-12, LP-13 and LP-14) has faster drug release rate than formulations containing 4.76 ml of water per 20 g of Liqui-Mass admixture (LP-9, LP-10 and LP-11). The difference factor (f_1_) and similarity factor (f_2_) for formulations containing the same concentration of Tween 80 and R-value, but with different water content shows differences in dissolution profile as shown in Table 4. This mathematical analysis, especially the f_1_ indicates that formulation containing 1.9 ml of water per 20 g of Liqui-Mass admixture have a faster drug release rate than 4.76 ml of water per 20 g of Liqui-Mass admixture. It also shows f_1_ values of these formulations are all above 15 and f_2_ values below or on the borderline of 50.

**Table 4:** Difference factor (f_1_) and similarity factor (f_2_) of formulation in comparison

Formulation in comparison	f_1_	f_2_
LP-9 (5 ml of water, 36% *w*/w tween 80 and R = 20:1) & LP-12 (2 ml of water, 36% *w*/w tween 80 and R = 20:1)	38.64	46.1
LP-10 (5 ml of water, 36% w/w tween 80 and R = 19:2) LP-13 (2 ml of water, 36% *w*/w tween 80 and R = 19:2)	33.01	50.21
LP-11 (5 ml of water, 36% w/w tween 80 and R = 6:1) & LP-14 (2 ml of water, 36% w/w tween 80 and R = 6:1)	32.18	48.87
LP-9 (5 ml of water, 36% w/w tween 80 and R = 20:1) & LP-10 (5 ml of water, 36% w/w tween 80 and R = 19:2)	7.45	85.06
LP-9 (5 ml of water, 36% w/w tween 80 and R = 20:1) & LP-11 (5 ml of water, 36% w/w tween 80 and R = 6:1)	20.6	66.13
LP-12 (2 ml of water, 36% w/w tween 80 and R = 20:1) & LP-13 (2 ml of water, 36% w/w tween 80 and R = 19:2)	3.03	93.12
LP-12 (2 ml of water, 36% w/w tween 80 and R = 20:1) & LP-14 (2 ml of water, 36% w/w tween 80 and R = 6:1)	8.17	75.82

On analyzing formulation with varying R-values, only formulation LP-9 (4.76 ml of water per 20 g Liqui-Mass admixture, 36% *w*/w Tween 80 and **R = 20:1**) and LP-11 (4.76 ml of water per 20 g Liqui-Mass admixture, 36% w/w Tween 80 and **R = 6:1**) shows a significant difference in dissolution profile according to f_1_ (20.6). Despite only LP-9 and LP-11 showing a significant difference in dissolution profile, the dissolution curve (Fig. 3) indicates that almost all of the formulations show a general trend that reduction in R-value increases the dissolution rate. This could be due to an increase in the concentration of Aerosil 300, which is hydrophilic, hence, improving water penetration into the pellet and promoting disintegration, which in turn enhances drug release rate. It is already known from the previous study that water content can significantly influence the dissolution rate [3]. The f_1_ increases and f_2_ decreases at a greater extent as the ratio between the carrier and coating material further increases. This indicates that the dissolution profiles become more different with a greater extent of differences in the ratio of carrier and coating material.

Overall it seems that R-value may have some effect on the drug release rate; however, the effect is small as revealed by the mathematical analysis (Table 4). The effect of R-value on dissolution rate is less obvious than water content or Tween 80 (co-solvent) concentration. In our previous study, the water content and Tween 80 concentration have a significant impact on the dissolution rate [3]. Although the R-value may not have as much impact on drug release rate as other parameters such as water content and Tween 80 concentration, it is still prudent to understand its effect on drug release to aid optimization of future Liqui-Pellet formulation.

**Fig. 4:**
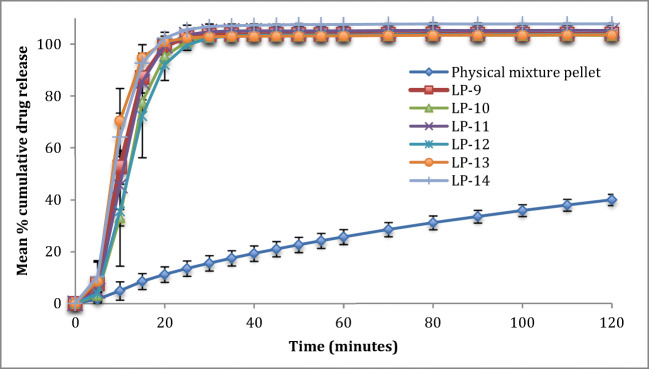
Dissolution profile of all successful Liqui-Pellet formulation containing 25 mg of naproxen in capsule (pH 7.4)

The drug release data of all successful Liqui-Pellet formulations show a fast dissolution rate at pH 7.4 (Fig. 4). All of these formulations start/nearing to plateau after ~25 min. The reason for the fast dissolution is due to the weak acidic property of naproxen, which makes it more soluble in alkaline than the acidic environment. It is advantageous for weakly acidic drugs to be in Liqui-Pellet dosage form. This is because Liqui-Pellet is usually small (under 2 mm in size) as seen in Fig. 1 and previous studies [1–3], which mean quick gastric emptying of Liqui-Pellet or quick arrival to the more alkaline small intestine, where weakly acidic drugs have better solubility, resulting in a potential for enhanced bioavailability.

### XRPD studies

**Fig. 5:**
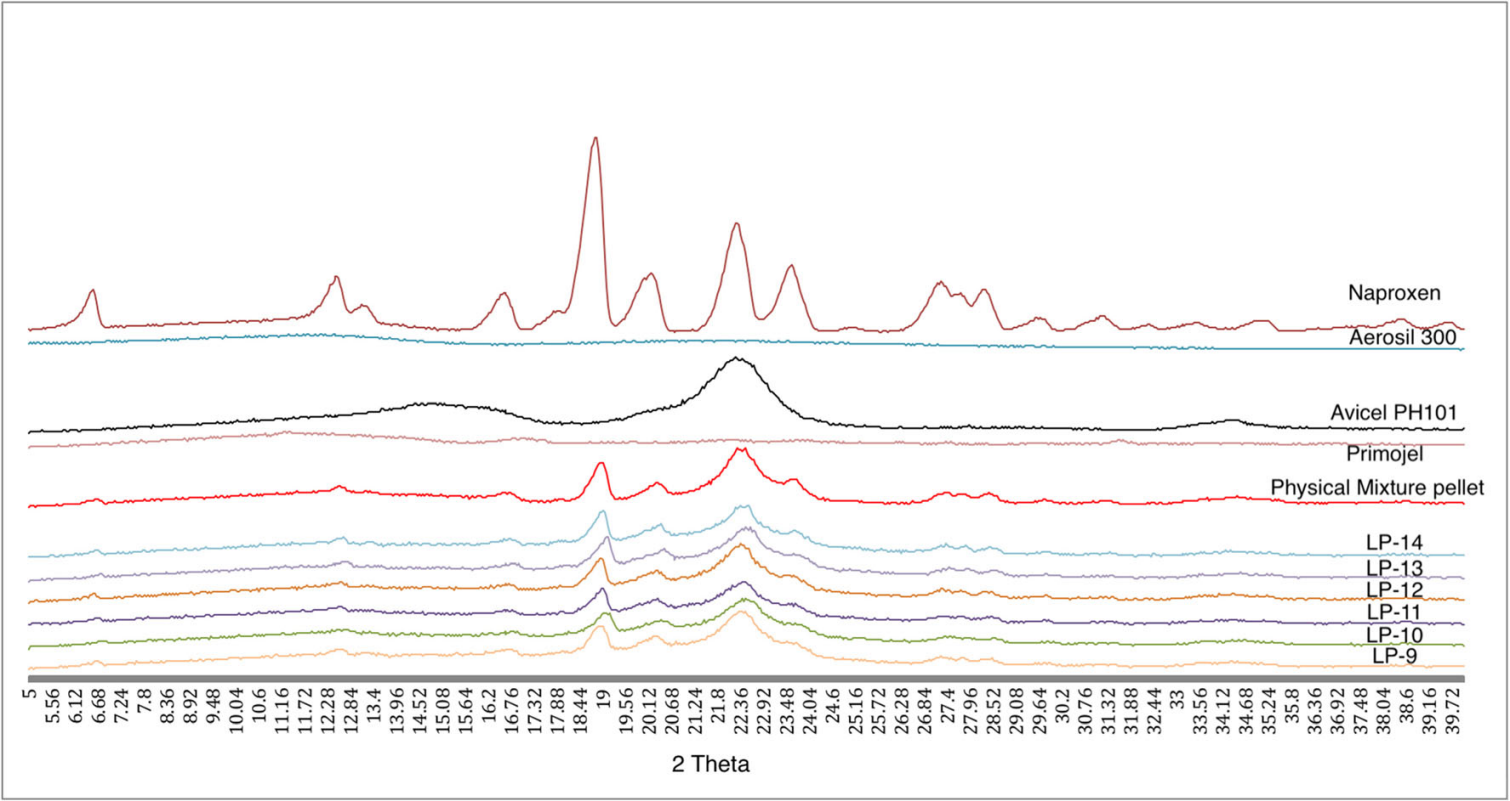
Diffraction peaks of naproxen, avicel, aerosil, primojel, physical mixture pellet and formulation LP-9 to LP-14

The XRPD results shows that naproxen peak at 2θ values of 6.4, 12.28, 12.96, 16.32, 18.72, 19.88, 22.16, 23.40, 26.96 and 28.04 (Fig. 5). This is similar to the naproxen peak from the authors’ previous studies [1–3]. The peak in the physical mixture only corresponds to naproxen and Avicel PH101, denoting that there is no interaction between naproxen and the excipients. Figure 5 does not show a big difference in XRPD between the physical mixtures and Liqui-Pellet formulations. This could be due to the presence of a high concentration of amorphous Avicel PH101 overshadowing the overall XRPD peaks.

A closer inspection is carried out in investigating the relative crystallinity. This is achieved through comparative analysis with respect to AUC at 18.9 ^o^ peak (Table 5). It should be pointed out that the data does not represent the whole percentage of crystallinity of each formulation because only one crystalline peak is used for analysis. The results show that physical mixture pellet and all the selected Liqui-Pellet formulations (LP-9 to LP-14) have reduced crystallinity in comparison to pure naproxen. All of the selected Liqui-Pellet formulations show lower % relative crystallinity compared to physical mixture pellet. This is expected as Liqui-Pellet contains a liquid vehicle, which solubilizes the API or holds the API in a molecularly dispersed state; hence, the crystallinity of API is reduced as shown in Fig. 5 and Table 5.

**Table 5:** Relative crystallinity in respect to AUC at 18.9 ^o^ peak among physical mixture pellet, LP-9 to LP-14

Formulation	% relative crystallinity via integrated peak area method	% relative crystallinity via peak height method
Physical mixture	12.92	20.60
LP-9	9.69	13.30
LP-10	8.08	9.50
LP-11	5.00	12.03
LP-12	6.31	14.88
LP-13	6.32	15.26
LP-14	8.01	15.80

### DSC studies

**Fig. 6:**
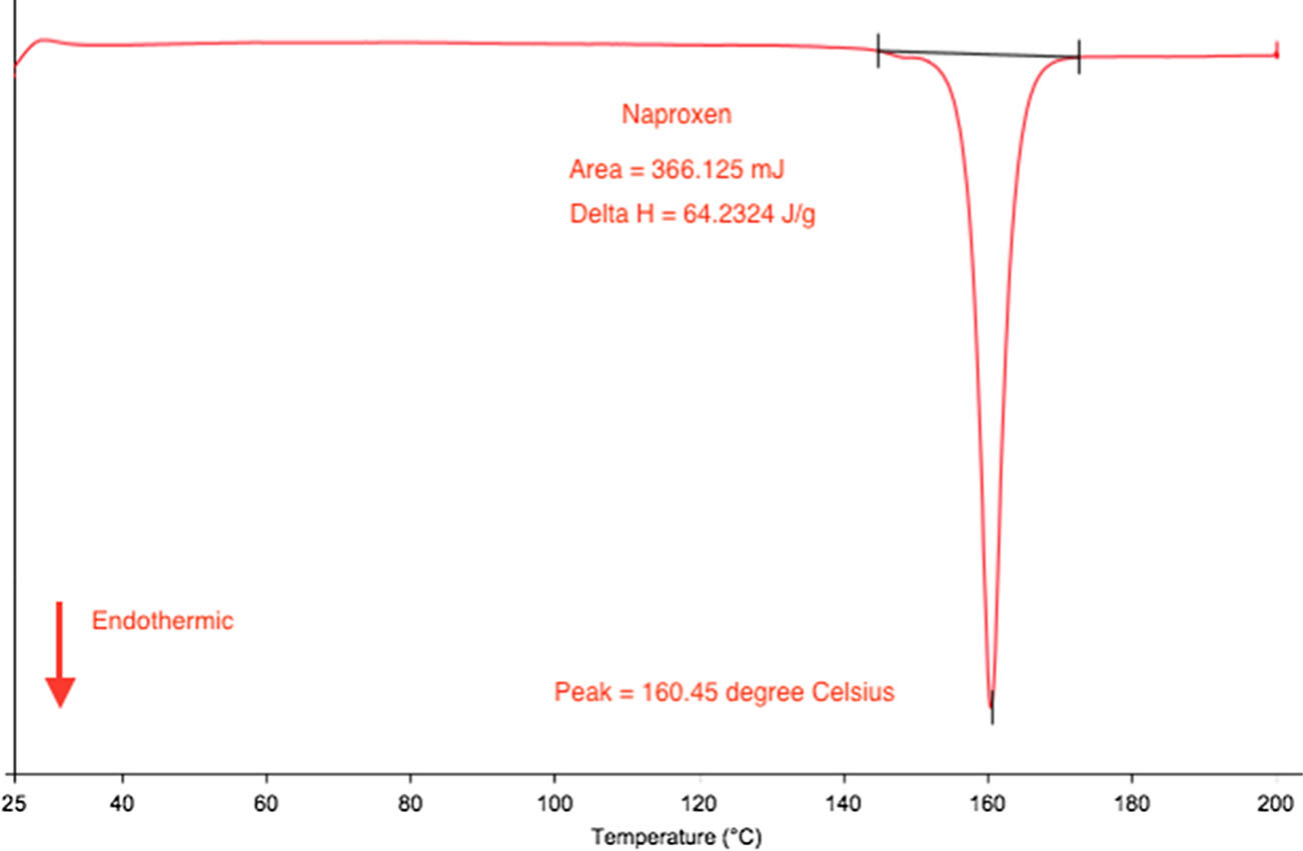
DSC thermograms of naproxen

**Fig. 7:**
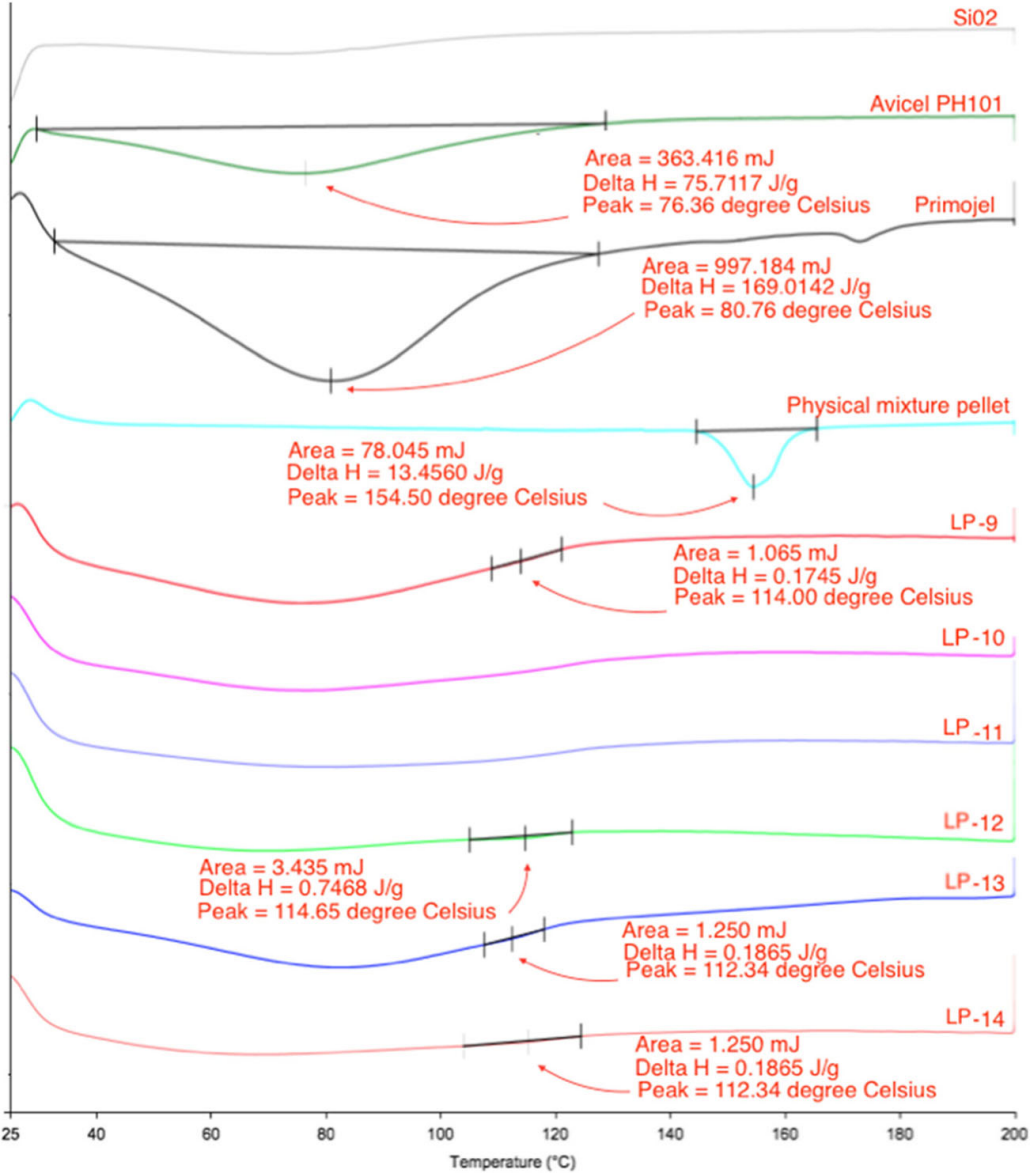
DSC thermograms of Aerosil 300, Avicel PH101, Primojel, physical mixture and all successful Liqui-Pellet formulations

All successful Liqui-Pellet formulations have shown reduced crystallinity. The naproxen crystalline state is presented as a sharp endothermic peak (T_m_ = 160.45 °C and ΔH = 64.23 J/g), which can be seen in Fig. 6. Avicel PH101 (T_m_ = 76.36 °C and ΔH = 80.73 J/g) and Primojel (T_m_ = 79.76 °C and ΔH = 257.79 J/g) present broad peaks (Fig. 7), which could be due to water evaporation from these hygroscopic excipients. This observation is also seen in Tiong et al. studies [29] and the authors’ previous studies [1, 2]. The amorphous Aerosil 300 does not have a definitive peak.

On analyzing naproxen and physical mixture pellet DSC endothermic peak (Fig. 7), there is a small shift of peak from 160.45 °C to 154.50 °C respectively, which may be due to Avicel PH101 influencing the overall peak of naproxen in the physical mixture pellets. Nonetheless, the crystalline state of naproxen is still present in the physical mixture. On comparing the physical mixture pellet, which has a definitive naproxen crystalline peak, to the successfully produced Liqui-Pellet formulations, the Liqui-Pellet formulation crystalline peak is largely reduced. This indicates the reduction of crystallinity, which is typical in liquisolid formulation, where API is solubilized or in a molecularly dispersed state.

## Conclusion

The data obtained from this investigation suggest that the ratio of Avicel PH101 (carrier) and Aerosil 300 (coating material) does not have a major effect on the success of Liqui-Pellet production. Formulations containing different R-value had the same success rate of Liqui-Pellet production. Particle size studies suggest that R-value may affect Liqui-Pellet size at a particular water content level. Attempt to succeed failed formulation using a freezing technique such as freezer and liquid nitrogen has proven ineffective. Despite only two formulations (LP-9 and LP-11) with different R-values showing significant differences in the dissolution profile (f_1_ = 20.6), the general trend in drug dissolution test shows that increased Aerosil 300 and decrease Avicel PH101 ratio increases the dissolution rate. This could be due to an increase of the hydrophilic Aerosil 300, which enhances the penetration of water into Liqui-Pellet and promoting disintegration. This can result to increase in drug release rate. The f_1_ increases and f_2_ decreases to a greater extent as the ratio of carrier decreases and coating material increases. Such a trend indicates that the dissolution profiles become more different with a greater extent of differences in the ratio of carrier and coating material. Overall it seems that R-value may have some effect on the drug release rate. The effect of R-value on dissolution rate is less obvious than water content or Tween 80 (liquid vehicle) concentration as seen in previous studies. Nonetheless, it is still imperative to understand the effect of R-value parameter on the drug release rate in order to aid further optimization of future Liqui-Pellet formulation.
